# Association study of the trinucleotide repeat polymorphism within *SMARCA2 *and schizophrenia

**DOI:** 10.1186/1471-2156-7-34

**Published:** 2006-06-03

**Authors:** Sarojini Sengupta, Lan Xiong, Ferid Fathalli, Chawki Benkelfat, Karim Tabbane, Zoltan Danics, Alain Labelle, Samarthji Lal, Marie-Odile Krebs, Guy Rouleau, Ridha Joober

**Affiliations:** 1 Department of Human Genetics, McGill University, Montreal, Canada; 2 Douglas Hospital Research Centre, Montreal, Canada; 3 Department of Psychiatry, McGill University, Montreal, Canada; 4 Center for the Study of Brain Diseases, Notre Dame Hospital, Montreal, Canada; 5 Department of Psychiatry, University of Tunis, Tunis, Tunisia; 6 National Institute of Psychiatry, Budapest, Hungary; 7 Department of Psychiatry, University of Ottawa, Ottawa, Canada; 8 Service Hospitalo-Universitaire, Hôpital Sainte-Anne, Paris, France

## Abstract

**Background:**

Brahma (BRM) is a key component of the multisubunit SWI/SNF complex, a complex which uses the energy of ATP hydrolysis to remodel chromatin. BRM contains an N-terminal polyglutamine domain, encoded by a polymorphic trinucleotide (CAA/CAG) repeat, the only known polymorphism in the coding region of the gene (*SMARCA2*). We have examined the association of this polymorphism with schizophrenia in a family-based and case/control study. *SMARCA2 *was chosen as a candidate gene because of its specific role in developmental pathways, its high expression level in the brain and some evidence of its association with schizophrenia spectrum disorder from genome-wide linkage analysis.

**Results:**

Family-based analysis with 281 complete and incomplete triads showed that there is no significant preferential transmission of any of the alleles to the affected offspring. Also, in the case/control analysis, similar allele and genotype distributions were observed between affected cases (n = 289) and unaffected controls (n = 273) in each of three Caucasian populations studied: French Canadian, Tunisian and other Caucasians of European origin.

**Conclusion:**

Results from our family-based and case-control association study suggest that there is no association between the trinucleotide repeat polymorphism within *SMARCA2 *and schizophrenia.

## Background

The SWI/SNF complex is a 2MDa, multi-subunit complex that uses the energy of ATP hydrolysis to disrupt nucleosome structure thereby increasing accessibility of transcription factors to their specific sites on DNA and histones. It is well conserved, with homologous complexes isolated from yeast, Xenopus, Drosophila and mammals [1]. SWI/SNF complexes purified from mammalian cell lines have been found to be heterologous with respect to their subunit composition, containing between 9–12 subunits [2]. However, each complex contains Brahma (BRM), a DNA-dependant ATPase, or the related protein BRG1. The precise subunit composition of the complex appears to confer functional specificity via protein-protein interactions with specific transcription factors. BRM has been shown to be specifically required for activation by the androgen receptor [3]. In addition, BRM and not BRG1, interacts specifically with CBF-1 and ICD22, two components of the Notch signalling pathway, a pathway that controls cell fate commitment in several developmental processes [4]. Studies in mice have shown that high levels of BRM, in comparison to levels of BRG1, are present in brain tissue of the animals [5].

BRM is encoded by the *SMARCA2 *gene. It is a member of the large, diverse SMARC (SWI/SNF-related, matrix-associated, actin-dependant regulator of chromatin) family. The only polymorphism within the encoding region of this gene is a stable, polymorphic trinucleotide repeat. We have carried out a case/control and family-based study to examine the association of this polymorphism with schizophrenia (SCZ).

Investigating *SMARCA2 *as a candidate gene for SCZ, is plausible for several reasons. First, genome-wide expression analysis of a post-mortem section of the dorsolateral prefrontal cortex of schizophrenia patients showed that *SMARCA2 *(also referred to as *hSNFa*) was upregulated 1.42-fold relative to non-affected controls [6]. Even though this upregulation of *SMARCA2 *has not been confirmed in other studies, several groups have demonstrated significant differences in mRNA levels in schizophrenic patients relative to controls. Gene expression profiles using post-mortem brain tissue (entorhinal cortex layer II stellate neurons) showed that 14% of the genes were up-regulated while 9% were down-regulated more than two-fold in the SCZ group relative to controls [7]. These results could be an indication that a global regulator of transcription may be involved in SCZ. BRM is one such global transcription regulator. BRM appears to be involved in the regulation of transcription (activation as well as repression) of a subset of genes. Genome-wide analysis, following deletion of SWI2 (the yeast homolog of BRM), showed that 1% of the 6,000 genes tested displayed more than a 3-fold change in mRNA levels, with some genes being up-regulated and others down-regulated [8,9].

A second reason why an association between BRM and schizophrenia is plausible is that BRM appears to play an important role in cell development in general, and neuronal differentiation in particular and SCZ is believed to be a neurodevelopmental disorder [10]. Unlike BRG1, where levels are relatively constant in all cells, the cellular concentration of BRM increases during cell differentiation. Studies with neural cell cultures showed that BRM expression increased during differentiation from neural precursor cells to neural cells [11]. Kondo and Raff [12] provide evidence that the conversion of oligodendrocyte precursor cells to neural stem cells required the recruitment of BRM. Further, BRM has been shown to strongly associate with CBF-1 and ICD22, two components of the Notch signalling pathway which play a key role in cell fate commitment during development [4].

Finally studying BRM as a candidate gene is supported by some linkage studies. *SMARCA2 *is located on chromosome 9p22.3. Genome scan meta analysis indicated that region 9p22.3-21.1 demonstrated significant linkage (*p *< 0.01) with a schizoaffective-bipolar and bipolar-I disease model [13].

## Results and discussion

**Figure 1:**
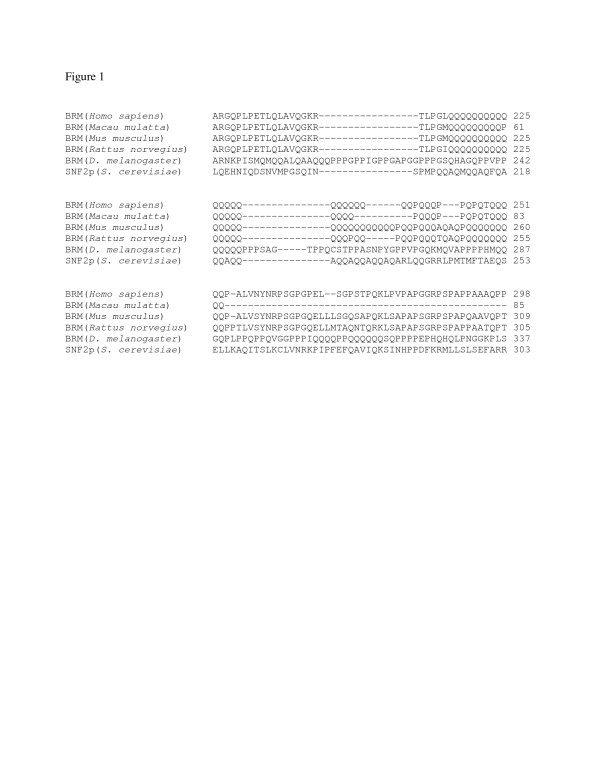
CLUSTAL W (1.82) multiple sequence alignment of the polyglutamine domain of mammalian BRM with the P/Q rich region in *D. melanogaster *and *S. cerevisiae*. Sequence alignment between BRM from *Homo sapiens *[GenBank: NP_003061.3], *Macau mulatta *[GenBank: AAV67666.1], *Mus musculus *[GenBank: NP_035546.2], *Rattus norvegius *[GenBank: NP_001004446.1], *D. melanogast*er [GenBank: P25439] and the homologous SNF2p from *S. cerevisiae *[GenBank: NP_014933.1] was conducted.

BRM encapsulates several distinct domains including a polyglutamine domain, the DNA-dependant helicase domain and the bromodomain. The N-terminal polyglutamine domain is well conserved in mammals with glutamine-rich regions also present in the homologous Drosophila Brahma and *Saccharomyces cerevisiae* Swi2/Snf2 proteins (Figure 1). BRM is well conserved with the only known polymorphism within the coding region of the gene *(SMARCA2) *being a trinucleotide repeat (CAA/CAG) polymorphism. The most commonly occurring allele, corresponding to 32 trinucleotide repeats (181 bps, according to Genotyper^® ^analysis) was designated the "0" allele. The two next most commonly occurring alleles were designated -1 (178 bps) and +1 (184 bps). Rare alleles -7, -5, +2, +3, and +4, having an allele frequency less than 0.01, were also obtained. Due to the problems that can arise in the cross-tabulation, since these cells had less than 5 cases each, the alleles were grouped for the statistical analyses- alleles -7, and -5 were grouped with the -1 allele and +2, +3, and +4 were grouped with the +1 allele.

Case/control analysis was carried out to compare the allele and genotype frequency of the trinucleotide repeat polymorphism within three Caucasian populations: French Canadian, Tunisian and other Caucasians of European origin. Similar allele and genotype distributions were observed between affected cases and unaffected controls in each of these three populations (Tables 3 and 4). The alleles in the affected and non-affected populations were in Hardy-Weinberg equilibrium, calculated by considering each allele in combination with every other allele. The family-based association analysis (bi-allelic mode; additive model) showed that none of the alleles were preferentially transmitted/under-transmitted to the affected offspring (Table 5). A minor under-transmission of the -1 allele (*p *= 0.034) was observed. However this effect was not significant when corrected for multiple testing.

**Table 3:** Trinucleotide repeat polymorphism allele frequency, comparing cases and controls within each of the three populations tested

Allele	French-Canadian		Tunisian		Caucasian of European origin	
	Case (n = 100)	Control (n = 102)	Case (n = 67)	Control (n = 46)	Case (n = 122)	Control (n = 125)

-1	0.11	0.15	0.1	0.11	0.08	0.1
0	0.87	0.85	0.86	0.84	0.91	0.88
+1	0.03	0.01	0.04	0.05	0.01	0.02

	*χ*^2 ^= 3.9, *df *= 2, *p *> 0.1		*χ*^2 ^= 0.2, *df *= 2, *p *> 0.5		*χ*^2 ^= 1.29, *df *= 2, *p *> 0.5	

**Table 4:** Trinucleotide repeat polymorphism genotype frequency, comparing cases and controls within each of the three populations tested

Genotype	French-Canadian		Tunisian		Caucasian of European origin	
	Case (n = 100)	Control (n = 102)	Case (n = 67)	Control (n = 46)	Case (n = 122)	Control (n = 125)

-1,0	0.21	0.25	0.19	0.2	0.15	0.18
0,0	0.74	0.75	0.73	0.7	0.83	0.78
0,+1	0.05	0.01	0.07	0.11	0.02	0.04

	*χ*^2 ^= 3.02, *df *= 2, *p *> 0.1		*χ*^2 ^= 0.41, *df *= 2, *p *> 0.5		*χ*^2 ^= 0.91, *df *= 2, *p *> 0.5	

**Table 5:** Transmission Disequilibrium Test and FBAT analysis

Allele	Allele Frequency	No. of informative triads	Z statistic	*p*
-1	0.12	53	-2.12	0.034
0	0.85	67	1.65	0.099
+1	0.03	23	0.35	0.72

These results suggest that there is no association between the trinucleotide repeat polymorphism within *SMARCA2 *and schizophrenia. Although all subjects included in the study were carefully diagnosed according to DSM criteria, as having schizophrenia or schizoaffective disorder, one cannot exclude the phenotypic diversity that is inherent to the disorder. For example, within our French Canadian group of affected subjects, 70.7% were diagnosed with paranoid schizophrenia, 5.05% with the disorganized type, 2.02% with schizoaffective disorder and 22.22% with undifferentiated schizophrenia. There has been considerable discussion recently that in studying the genetics of schizophrenia and complex psychiatric disorders, in general, that it is necessary to examine suitable endophenotypes within the disorder rather than to examine the disorder as a whole [17-19]. It remains important therefore to examine the association of this trinucleotide repeat polymorphism within *SMARCA2 *with specific endophenotypes resulting from neurodevelopmental deficits, given the importance of BRM in development and neural development in particular. Also given the minor effect detected with the family-based association study, it may be important to re-examine this polymorphism with a larger sample size.

This study also offers a step forward in understanding the polyglutamine domain within BRM. Although BRM has been shown to be important in transcriptional regulation, little is known about the role of the polyglutamine domain. In the populations we have studied, we have not observed an expansion of the trinucleotide repeat region, even though the number of glutamines (32) is close to the pathogenic threshold of ~35–40 glutamines [20]. The trinucleotide repeat appears therefore to be stable, though polymorphic. We sequenced the trinucleotide repeat in six individuals, two each being homozygous for the -1, 0 and +1 alleles. The sequence encoded by this domain, in the 0 allele, is -**Q**_23_-**P**- **Q**_3 _-**P**_2 _-**Q**- **P**-**Q**-. The sequence of the -1 allele was -**Q**_22 _-P- Q_3 _-P_2 _-Q- P -Q- while the sequence of the +1 allele was -**Q**_24 _-P- Q_3 _-P_2 _-Q- P -Q-. It therefore appears that the terminal CAG repeat in the first block of CAA/CAG repeats is the polymorphic site, at least in the individuals studied. This is consistent with results obtained in examining the different alleles at this locus in an Indian population [21]. Further functional studies would be important to assess the role of this polyglutamine domain in protein-DNA and protein-protein interactions. This may be informative particularly since this domain appears to be a conserved domain in BRM, which itself is a well conserved regulator of transcription.

## Conclusion

Results from our family-based and case-control association study suggest that there is no association between the trinucleotide repeat polymorphism within *SMARCA2 *and schizophrenia.

## Methods

### Subjects

Subjects diagnosed with schizophrenia or schizoaffective disorder, their families and unrelated healthy controls were recruited from three populations: Tunisian, French Canadian and other Caucasians of European origin. Patients were diagnosed using DSM-III-R or DSM-IV criteria on the basis of the Diagnostic Interview for Genetic Studies (DIGS) and complementary data from the medical files. Most of these subjects had previously participated in a pharmacogenetic study with a detailed evaluation of therapeutic response to medication [14]. For the family-based study, subjects and their families were recruited from mental health facilities in Montreal, Ottawa, Hungary, France, and Tunisia. A total of 281 complete (n = 182) and incomplete (n = 99) triads were genotyped (Table 1).

**Table 1:** Summary of subjects used in the family-based study

	No of families			No of triads		
	Families with one affected individual	Families with more than one affected individual	Total	Complete triad	Incomplete triad*	Total

French Canadian	41	0	41	13	28	41
Tunisian	24	12	36	52	2	54
Other Caucasian of European origin-1	58	1	59	28	35	63
Other Caucasian of European origin-2	113	5	118	89	34	123

Total	236	19	255	182	99	281

* Incomplete triads include families with missing parent(s).

"Other Caucasian of European origin-1" included Caucasian triads (excluding French Canadian triads) recruited from Montreal and Ottawa.

"Other Caucasian of European origin-2" included Caucasian triads recruited from Hungary and France.

Case/control analysis was conducted exclusively with subjects from Montreal, Ottawa and Tunisia since unaffected control subjects were not available for analysis from Hungary and France. This step was undertaken to minimize any effects arising due to population stratification. Unrelated cases (n = 289) and controls (n = 273) were used for the case-control analysis. The cases included (Table 2): (1) unrelated probands (n = 123), recruited from Montreal, Ottawa, and Tunisia, who were included in the family-based analysis. Only probands from families having one affected individual were included for this case-control analysis (Table 1), in order to avoid introducing bias by selecting between multiple affected individuals from a single family; (2) subjects (n = 166) recruited from mental health facilities in the three regions, for whom parent DNA samples were not available (termed "isolated cases"). Table 2 gives a detailed break-up of the ethnicities of the cases and controls.

**Table 2:** Summary of subjects used in the case-control analysis

	Case			Control
	Cases included in family-based study*	Isolated cases^†^	Total	

French Canadian	41	59	100	102
Tunisian	24	43	67	46
Other Caucasian of European origin-1	58	64	122	125
Total	123	166	289	273

* Subjects from families having more than one affected individual were not included in the case/control study (please refer to Table 1). Only unrelated cases and controls were used for this analysis.

^† ^Isolated cases were those subjects for whom parent DNA samples were not available.

Controls were recruited in Montreal, Ottawa and Tunisia, either by newspaper advertisement or random sampling from a downtown population or open advertisement among the employees of the hospitals where the studies were conducted. Each control subject was evaluated by a trained clinician using the Structured Clinical Interview for DSM-IV, Non-patient edition (SCID-NP) to ensure that the individual did not have an ongoing or previous psychiatric illness. Written, informed consent was obtained from each patient and non-affected subject prior to beginning the study. The research protocol and the consent forms were approved by the Ethics Committee of each Institutional Review Board.

### Genotyping

Genomic DNA was extracted from peripheral blood using standard procedures. The trinucleotide repeat within *SMARCA2 *was amplified by polymerase chain reaction (PCR) using the following primers: forward-5'TGCAGTCCAGGGGAAAAGGACGTT3', reverse-5'CCCGGGCCAGATGGTCTGTTGTAG3'. The forward primer was fluorescent labeled with 6-FAM™ (Applied Biosystems). A 15 μl PCR reaction, containing 10 ng DNA, 2.5 mM MgCl_2_, 0.25 mM of each dNTP, 0.33 μM of each primer, and 0.6 units Taq Polymerase was carried out. After initial denaturation at 94°C for 5 minutes, 35 cycles were carried out at 94°C for 1 min, 61.3°C for 1 min, 72°C for 1 min, followed by a final extension at 72°C for 30 min.

After verification of the PCR amplification by agarose gel electrophoresis, 0.5 μls of each sample was mixed with 0.3 μls size standard (GeneScan™ 500 LIZ™) and 9.2 μls deionized formamide (Applied Biosystems), denatured at 95°C for 5 minutes, and placed on ice. Each 96-well plate was analyzed using the ABI PRISM^® ^3100 Genetic Analyzer and GeneScan v3.7. The sizes of the DNA fragments were obtained in comparison to the DNA standard using Genotyper^® ^Software v3.7. The most frequently occurring allele, corresponding to a size of 181 bp (obtained using Genotyper^®^) was designated "0". The sizes of each of the respective alleles increased or decreased proportionately by 3 bps i.e. alleles -1, and +1 had sizes of 178 and 184 bps respectively.

### Statistical analysis

The Transmission Disequilibrium Test [15] was conducted using the FBAT (Family-Based Association Tests) program [16]. Allele frequencies in affected subjects and non-affected controls were compared using the Pearson chi-square (*χ*^2^) statistic in Statistica^®^. Statistical significance was established at *p *< 0.05.

## Abbreviations

BRM – Brahma

DIGS – Diagnostic Interview for Genetic Studies

FBAT – Family-Based Association Tests

PCR – polymerase chain reaction

SCID-NP – Structured Clinical Interview for DSM-IV, Non-patient edition

SCZ – schizophrenia

SMARC – SWI/SNF-related, matrix-associated, actin-dependant regulator of chromatin

## Authors' contributions

SS, LX, FF performed the data analysis. SS drafted the manuscript. CB, KT, ZD, AL, SL, MK, GR and RJ were involved in the study design and provided clinical support. RJ was responsible for the conception of the study, supervision of the research project and drafting of the manuscript.
